# Combined treatment with C16 peptide and angiopoietin-1 confers neuroprotection and reduces inflammation in 3-nitropropionic acid-induced dystonia mice

**DOI:** 10.18632/aging.203354

**Published:** 2021-07-29

**Authors:** Xiao-Xiao Fu, Hua-Ying Cai, Hong Jiang, Shu Han

**Affiliations:** 1 Institute of Anatomy and Cell Biology and Sir Run Run Shaw Hospital, Medical College, Zhejiang University, Hangzhou, China; 2 Department of Neurology, Sir Run Run Shaw Hospital, Medical College, Zhejiang University, Hangzhou, China; 3 Department of Electrophysiology, Sir Run Run Shaw Hospital, Medical College, Zhejiang University, Hangzhou, China

**Keywords:** 3-Nitropropionic acid, dystonia model, C16, Ang-1, improving micro-environment

## Abstract

Dystonia is a disorder associated with abnormalities in many brain regions including the basal ganglia and cerebellum. The toxin 3-Nitropropionic acid (3-NP) can induce neuropathologies in the mice striatum and nigra substance, including excitotoxicity, neuroinflammation, and extensive neuronal atrophy, characterized by progressive motor dysfunction, dystonia, and memory loss, mimicking those observed in humans. We established a mouse model of dystonia by administering 3-NP. Given the reported neuroprotective effects of the endothelial growth factor angiopoietin-1 (Ang-1) and the anti-inflammatory integrin αvβ3 binding peptide C16, we performed this study to evaluate their combined effects on 3-NP striatal toxicity and their therapeutic potential with multiple methods using an *in vivo* mouse model. Sixty mice were equally and randomly divided into three groups: control, 3-NP treatment, and 3-NP+C16+Ang-1 treatment. Behavioral and electrophysiological tests were conducted and the effect of the combined C16+Ang-1 treatment on neural function recovery was determined. We found that C16+Ang-1 treatment alleviated 3-NP-induced behavioral, biochemical, and cellular alterations in the central nervous system and promoted function recovery by restoring vascular permeability and reducing inflammation in the micro-environment. In conclusion, our results confirmed the neuroprotective effect of combined C16+Ang-1 treatment and suggest their potential as a complementary therapeutic against 3-NP-induced dystonia.

## INTRODUCTION

The neurological movement disorder, dystonia, is associated with abnormalities in various regions of the brain including the basal ganglia and cerebellum. Dystonia is characterized by involuntary repetitive contractions of antagonistic muscle groups that disrupt motor signals between the brain and neck, resulting in twisting movements and abnormal postures [1–3]. The different types of dystonia vary in etiology and phenotype, and symptoms can range from mild to severe [4, 5]. This disorder can occur as early as in childhood and its pathophysiology includes neuronal damage mainly in the striatum and nigra substance, as well as lesions in other regions of the brain [6]. Causative factors of dystonia include infection, brain trauma, stroke or hypoxia, exposure to heavy metals or carbon monoxide, and adverse drug reactions. Neurotransmitter imbalance in the basal ganglia, excitotoxicity, oxidative stress, mitochondrial dysfunction, and neuroinflammation [7] have been postulated as possible underlying mechanisms for dystonia [2, 8]. As treatment options for dystonia are mainly symptomatic [8, 9], lack efficacy, and give rise to systemic toxicity [9], there is an imperative need for the development of new and effective alternative treatments as well as a cure.

Animal models of progressive neurodegenerative diseases like Huntington’s disease (HD) can be established with 3-Nitropropionic acid (3-NP) treatment. 3-NP is an irreversible inhibitor of succinate dehydrogenase, an enzyme located in the inner mitochondrial membrane, which inhibits the Krebs cycle and the electron transport chain [10]. Locomotor hypoactivity, dystonia, chorea, dyskinesia, progressive memory loss, behavioral changes, increased inflammation and apoptosis, and striatal lesions characteristic of motor neuron disorders, and other central nervous system (CNS) disorders [8] have been induced by 3-NP in such animal models as primates and mice [11–13]. Given the parallels in dystonia pathophysiology between humans and animals, insights garnered from this study would be applicable to the development of novel therapies.

Induction by 3-NP stimulates the release of pro-inflammatory cytokines, thereby leading to microglia activation, excitotoxicity, neuroinflammation, and CNS disorders [7]. Inflammatory cell infiltration in the CNS due to increased permeability of the blood-brain barrier can exacerbate neuroinflammation and neuronal damage. Preventing microglial activation and reducing neuroinflammation conferred neuroprotection in 3-NP animal models [7, 14]. Thus, targeting inflammation could be a promising approach in therapeutic interventions.

The endothelial growth factor, angiopoietin-1 (Ang-1) acts through the receptor tyrosine kinase Tie2, while C16 (KAFDITYVRLKF) binds to αvβ3 and α5β1 integrins to promote the survival of endothelial cells in blood vessels. By specifically recognizing and binding to αvβ3 integrins, C16 can competitively inhibit leukocyte-endothelial interactions and inflammatory cell transmigration into the CNS [15, 16]. C16 and Ang-1 administration can protect against vascular leakage, inflammation, and axonal loss in experimental autoimmune encephalomyelitis [17–19] and neuromyelitis optica [19]. Since neuroinflammation is a common occurrence in several neuronal degenerative diseases in the CNS, we designed this study to investigate the efficacy of a combination of Ang-1 and C16 peptide in neuroprotection in a 3-NP-induced dystonic mouse model. We assessed the behavior of the dystonic mice, inflammation, axonal loss, neuronal apoptosis, autophagy, and functional recovery using electrophysiology, immunohistochemistry, electron microscopy, and western blotting. Our findings provide novel insights into targeting and alleviating 3-NP-induced effects and could potentially be applied clinically in dystonia therapy.

## RESULTS

### C16+Ang-1 can improve 3-NP-induced functional impairment

**Figure 1:**
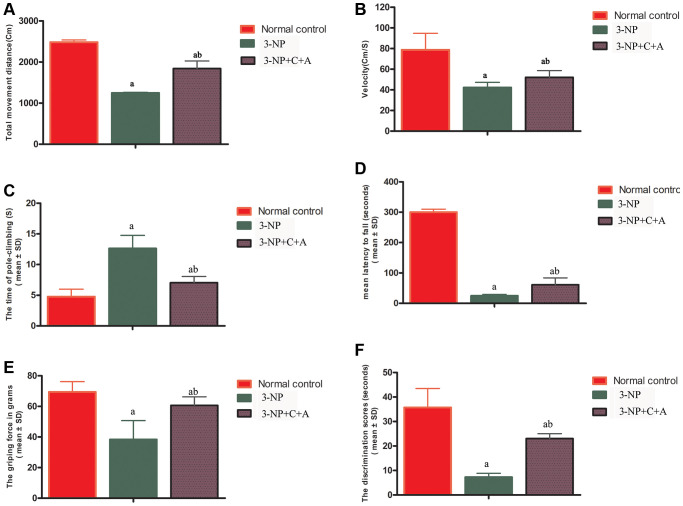
C16+Ang-1 treatment can alleviate 3-NP-induced motor functional impairment as shown by the open field (**A**–**B**), pole-climbing (**C**), rotorod (**D**), and gripping ability (**E**) tests, and memory impairment in recognition as evidenced by the novel object recognition test (**F**). Measurements of the (**A**) total distance travelled by the mice and (**B**) mean velocity of the control, 3-NP, and 3-NP+C16+Ang-1 groups in the open field test. Motor functional assessment revealed neurological disabilities with 3-NP insult following 3-NP treatment, while the 3-NP+C16+Ang-1-treated mice showed a reversal of these effects. (**C**) Measurements of the mean pole climbing time for the three groups. (**D**) In the rotarod test, 3-NP-treated mice remained on the device for a significantly shorter time compared to control mice, but 3-NP+C16+Ang-1 treatment inhibited this phenomenon. (**E**) The average forepaw grip strength of 3-NP-treated mice decreased significantly compared with that of controls but improved with 3-NP+C16+Ang-1 treatment. (**F**) The discrimination scores (%) for the three groups showed that 3-NP impaired the object discovery ability of the mice while 3-NP+C16+Ang-1 treatment alleviated the disorder. ^a^*P* < 0.05 versus control; ^b^*P* < 0.05 versus 3-NP-treated mice.

Mice exhibited functional impairment following 3-NP injections, evidenced by a significant decrease in the distance travelled (Figure 1A), mean velocity (Figure 1B) in the OFT, and prolonged mean pole climbing time (Figure 1C). In addition, 3-NP-treated mice (3-NP group) remained on the rotarod for a shorter period (Figure 1D). Weaker mean forepaw grip strength (Figure 1E) for mice in the 3-NP group was noted, indicating the loss of limb muscle strength. In the NOR test, the 3-NP-treated mice spent around the same time scrutinizing novel and familiar objects, indicating a failure of spatial cognition discrimination (Figure 1F). Compared to the 3-NP group, the 3-NP+C16+Ang-1 group showed improved motor functions, suggesting that C16+Ang-1 treatment could ameliorate 3-NP-induced effects on motor function.

### C16+Ang-l treatment can alleviate abnormal synchronous muscle contractions

**Figure 2:**
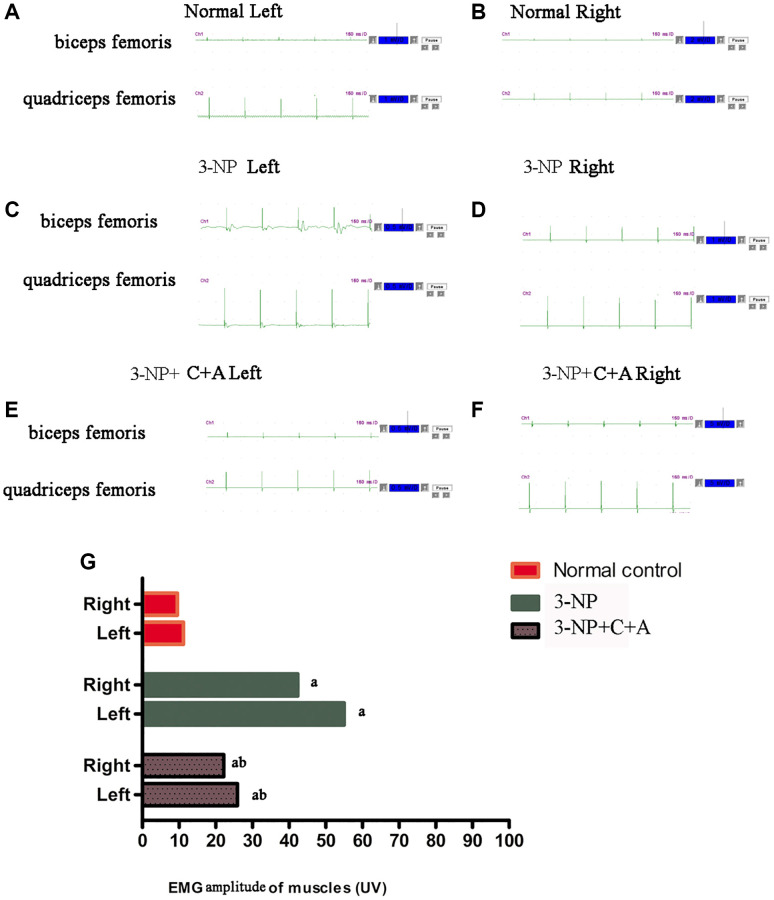
**C16 +Ang-l treatment can alleviate abnormal synchronous muscle contractions detected using electrophysiological methods.** (**A**–**F**) Muscle contraction wave profiles showing the amplitude (uV) of the (**A**, **B**) control, (**C**, **D**) 3-NP, and (**E**, **F**) 3-NP+C16+Ang-1 groups. Synchronous contraction of agonist (quadriceps femoris) and antagonist (biceps femoris) muscles indicated dystonia in the 3-NP group. This phenomenon of synchronous muscle contraction was alleviated by C16+Ang-1 treatment. (**G**) Quantification of the amplitude observed for each group. ^a^*P* < 0.05 versus control; ^b^*P* < 0.05 versus 3-NP-treated mice.

Dystonia is characterized by involuntary contractions of the agonist/antagonistic muscle pair, frequently causing twisting movements or abnormal postures. Upon stimulation, the agonist muscle (quadriceps femoris) in the control group was contracted while the antagonist muscle (biceps femoris) was relaxed (Figure 2A–2B). Synchronous contraction of agonist (quadriceps femoris) and antagonist (biceps femoris) muscles occurred upon 3-NP-induced dystonia, evidenced by the high amplitude observed for the wave profiles of both muscles (Figure 2C–2D). This phenomenon of synchronous agonist/antagonist muscle contraction was alleviated upon C16+Ang-1 treatment (Figure 2E–2F). Quantification of the amplitude observed for each group was showed in Figure 2G.

### Treatment with C16+Ang-l can suppress 3-NP-induced inflammation

**Figure 3:**
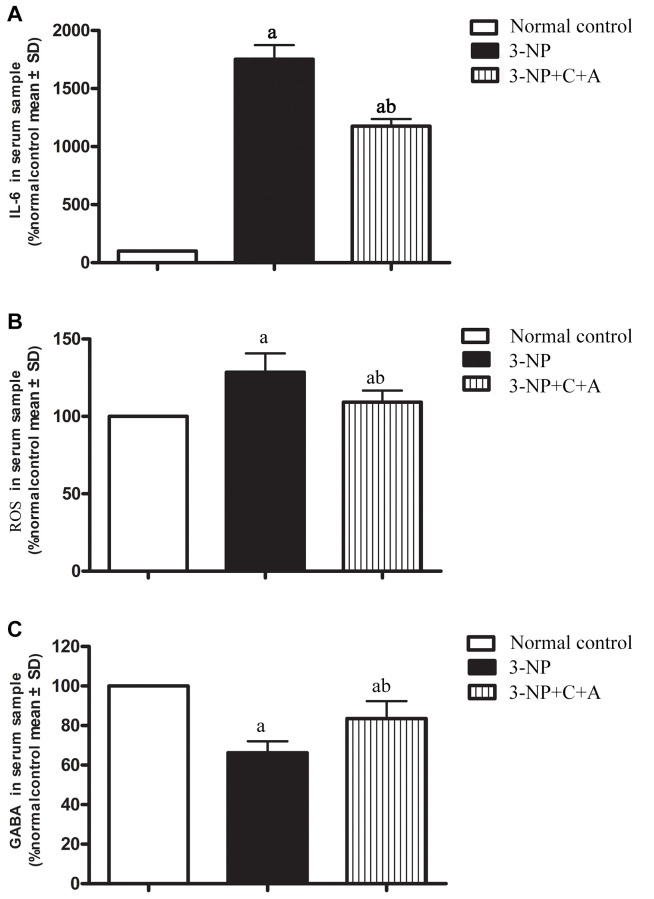
ELISA analysis of inflammatory cytokines (**A**) IL-6 and (**B**) ROS, as well as the inhibitory neurotransmitter. (**A**, **B**) The 3-NP+C16+Ang-1 group showed decreased IL-6 and ROS levels, suggesting that C16+Ang-1 could prevent 3-NP-induced inflammation. (**C**) GABA levels in serum samples of the control, 3-NP, and 3-NP+C16+Ang-1 groups. Suppression of inflammatory factors and increased GABA level could protect neurons that have been damaged by oxidative stress and excitatory toxin. ^a^*P* < 0.05 versus control; ^b^*P* < 0.05 versus 3-NP-treated mice.

Administration of 3-NP significantly elevated pro-inflammatory cytokine IL-6 levels in the 3-NP group (Figure 3A). Moreover, a high level of ROS indicative of oxidative stress was detected in the 3-NP group (Figure 3B). The 3-NP+C16+Ang-1 group showed decreased IL-6 and ROS levels, suggesting that C16+Ang-1 could prevent 3-NP-induced inflammation. Furthermore, ELISA showed that the GABA levels decreased following 3-NP treatment but increased following C16+Ang-1 treatment (Figure 3C).

**Figure 4:**
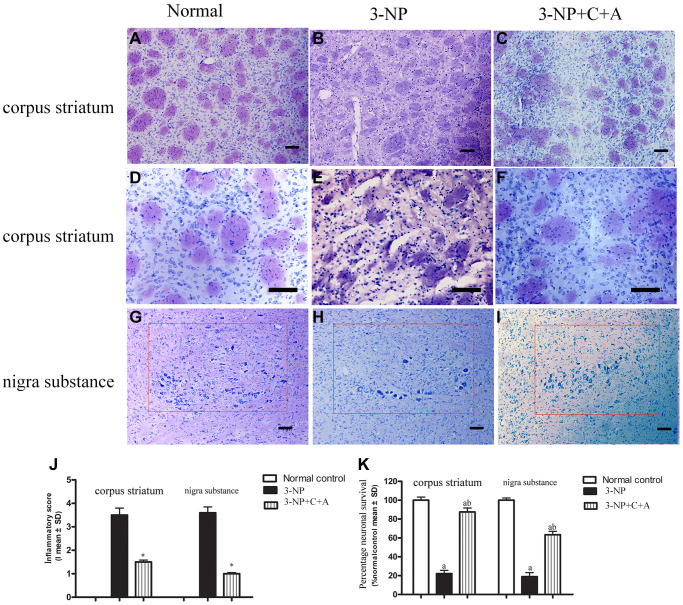
(**A**–**F**) HE staining showed inflammatory cells in the corpus striatum of each group at low magnification (×100, **A**–**C**) and higher magnification (×200, **D**–**F**); and (**G**–**I**) images of the surviving neurons within the nigra substance region (red box) for the control, 3-NP, and 3-NP+C16+Ang-1 groups. (**J**–**K**) Semi-quantitative profiles of the (**J**) inflammation scores (0, no inflammation; 1, cellular infiltrates only detected around meninges and blood vessels; 2, mild infiltrates detected in parenchymal tissues (1–10 inflammatory cells per slide); 3, moderate infiltrates observed in parenchymal tissues (11–100 inflammatory cells per slide); and 4, severe infiltrates in parenchymal tissues (>100 inflammatory cells per slide)) as well as the (**K**) neurons in each group visualized using a Nikon TE-300 microscope (Nikon, Japan) at ×200 magnification. The percentage (%) of neuronal survival was calculated with respect to the % of the sham control. Three randomly selected fields-of-view per section were selected for counting. The entire substantia nigra region comprising of surviving cells that were included in the count is also shown (within red box). Scale bar = 100 μm. ^a^*P* < 0.05 versus control; ^b^*P* < 0.05 versus 3-NP-treated mice. 3-NP, 3-NP; 3-NP+C+A, 3-NP+C16+Ang-1.

**Figure 5:**
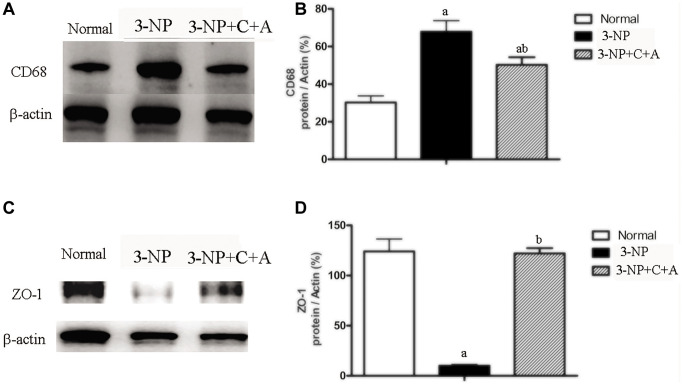
Western blot analyses of the levels of (**A**, **B**) CD68 (**C**, **D**) ZO-1 in the control, 3-NP, and 3-NP+C16+Ang-1 groups revealed that treatment with C16+Ang-l can suppress 3-NP-induced inflammation, protect the blood vessels and promote neuroprotection. Detection of CD68 indicated microglia/macrophage activation. Zonula occludens protein-1 (ZO-1) is a marker protein for tight junctions in epithelial cells. ZO-1 protein is often destroyed in 3-NP-induced dystonia. Scale bar = 100 μm. ^a^*P* < 0.05 versus control; ^b^*P* < 0.05 versus 3-NP-treated mice.

From HE staining, infiltration of inflammatory cells in the corpus striatum and nigra substance of the 3-NP group was observed, but inflammation was attenuated in the 3-NP+C16+Ang-1 group (Figure 4A–4F, 4J). Furthermore, western blot analysis showed upregulation of the inflammation marker CD68. Treatment with C16+Ang-1 could reduce the 3-NP-induced CD68 levels (Figure 5A, 5B), suggesting that C16+Ang-1 treatment could suppress inflammation.

### Treatment with C16+Ang-l can alleviate 3-NP-induced TH and CHAT expression decline, neuronal death, and synaptophysin loss

**Figure 6:**
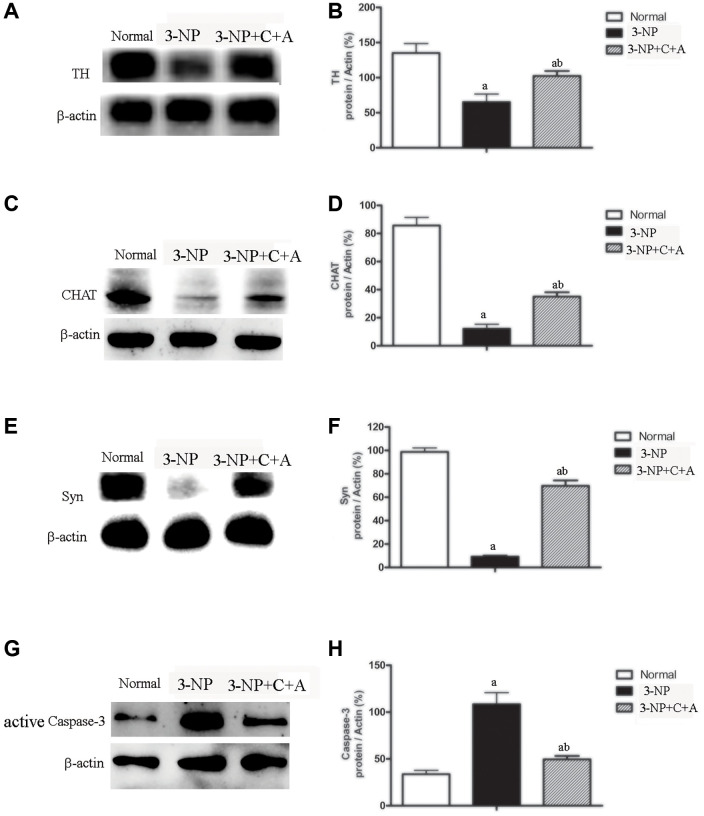
**Treatment with C16+Ang-l can alleviate 3-NP-induced TH and CHAT expression decline, neuronal death, and synaptophysin loss.** Western blot semi-quantitative analyses of (**A**–**B**) TH (for dopamine neurons), (**C**–**D**) CHAT (for cholinergic neurons), (**E**–**F**) Syn (synapse-associated proteins that showed synaptic plasticity and correlate with cognitive decline), and (**G**–**H**) active caspase-3 (an enzyme involved in mammalian apoptotic cell death) in the control, 3-NP, and 3-NP+C16+Ang-1 groups. ^a^*P* < 0.05 versus control; ^b^*P* < 0.05 versus 3-NP-treated mice.

**Figure 7:**
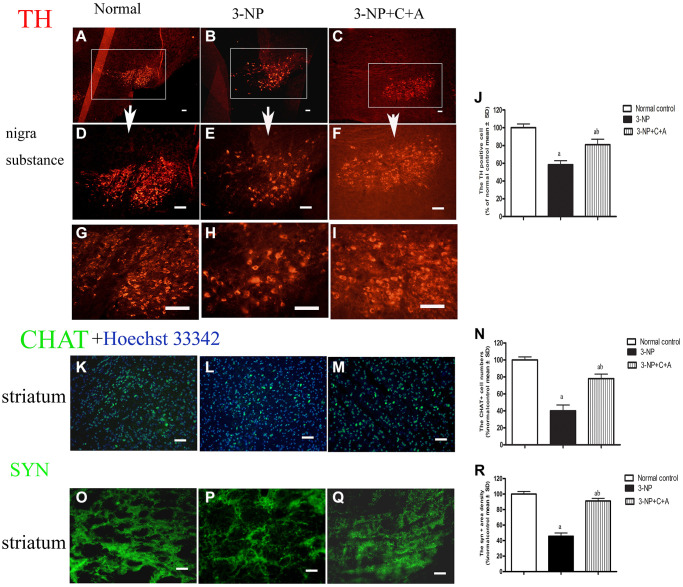
**Treatment with C16+Ang-l can alleviate 3-NP-induced TH and CHAT expression decline and synaptophysin loss.** Immunofluorescence images showing (**A**–**I**) TH-positive cells (red, dopaminergic neurons; white box in (**A**–**C**), nigra substance at low magnification ×40; (**D**–**F**) showed the same area at medium magnification ×100; and (**G**–**I**) at high magnification ×200), (**K**–**M**) CHAT-positive cells (green, cholinergic neurons; blue, Hoechst33342-stained cell nuclei), and (**O**–**Q**) the pixel of Syn-positive cells (synapse-associated proteins that showed synaptic plasticity and correlated with cognitive decline) in the control, 3-NP, and 3-NP+C16+Ang-1 groups. Scale bar = 100 μm. (**J**, **N**, **R**) semi-quantitative profiles of TH (**J**), CHAT (**N**) and Syn (**R**) in the control, 3-NP, and 3-NP+C16+Ang-1 groups. ^a^*P* < 0.05 versus control; ^b^*P* < 0.05 versus 3-NP-treated mice.

**Figure 8:**
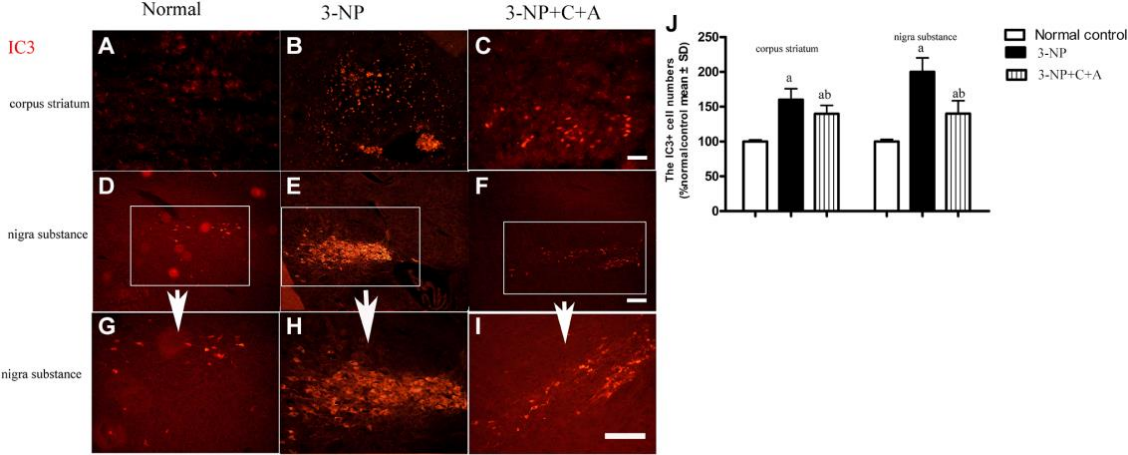
**Treatment with C16+Ang-l can alleviate 3-NP-induced neuronal loss.** Immunofluorescence staining in the (**A**–**C**) corpus striatum and (**D**–**F**) nigra substance (white box) at magnification ×100). (**G**–**I**) The same area of the nigra substance at ×200. (**J**) Semi-quantitative profiles of IC3 (an autophagy marker) in the control, 3-NP, and 3-NP+C16+Ang-1 groups. Scale bar = 100 μm. ^a^*P* < 0.05 versus control; ^b^*P* < 0.05 versus 3-NP-treated mice.

Dystonia is a central motor network disease characterized by a loss of cholinergic neurons and dopamine (DA) depletion accompanied by the loss of synapses. We assessed the expression of TH (marker for dopaminergic neurons), CHAT (marker for cholinergic neurons), Syn (marker for synapses), and active caspase-3 (an enzyme involved in the execution of the mammalian apoptotic cell death program) in the lysate of the corpus striatum and nigra substance tissues by western blotting (Figure 6A–6H). We found decreased levels of TH, CHAT, and Syn, but increased active caspase-3 levels in the 3-NP group. These effects were alleviated in the 3-NP+C16+Ang-1 group. HE staining also showed prominent neuronal loss, and these effects were inhibited in the 3-NP+C16+Ang-1 group (Figure 4G–4I, 4K). The results from the immunofluorescence staining of TH (Figure 7A–7J), CHAT (Figure 7K–7N), and Syn (Figure 7O–7R) confirmed the western blotting (Figure 6) and HE staining (Figure 4) results. Moreover, immunostaining showed that C16+Ang-1 treatment reduced the 3-NP-induced increase in the autophagy marker IC3 (Figure 8).

### Treatment with C16+Ang-1 reduces vascular leakage and blood-brain barrier permeability

**Figure 9:**
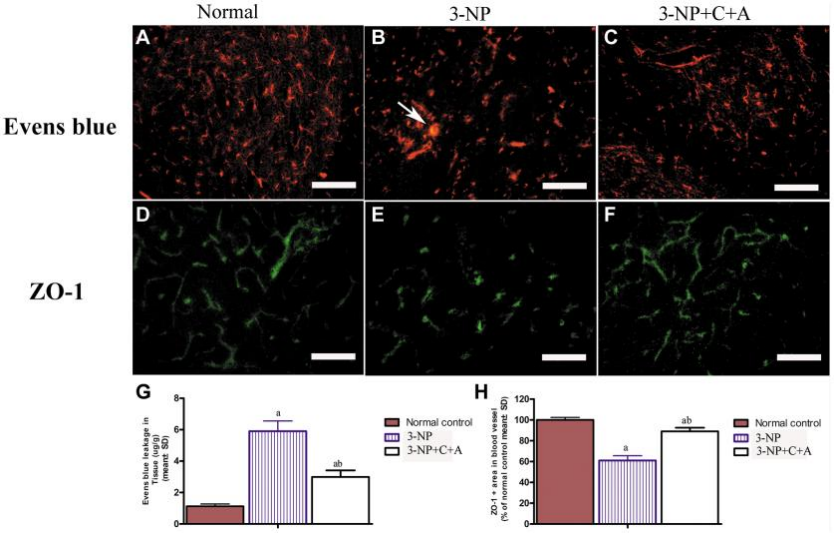
(**A**–**F**) Treatment with C16+Ang-l can alleviate 3-NP-induced permeability of the blood-brain barrier (leakage of Evans Blue dye, red) and loss of tight junctions (ZO-1 immunostaining, green) in the (**A**, **D**) control, (**B**, **E**) 3-NP, and (**C**, **F**) 3-NP+C16+Ang-1 groups. Scale bar = 100 μm. (**G**, **H**) Semi-quantitative profiles of (**G**) Evans Blue staining and (**H**) ZO-1 immunostaining. ^a^*P* < 0.05 versus control; ^b^*P* < 0.05 versus 3-NP-treated mice.

Evans Blue (EB) was injected into blood vessels to detect blood vessel leakage (to assess the degree of tissue edema) in each group. Detection was performed using a red laser at a wavelength of 405 nm. If EB leakage into tissue was absent, the red stain would only be detected within the blood vessel under the fluorescence microscope, whereas the tissues surrounding blood vessels would be stained red if there was EB leakage (due to the increased vascular permeability). In our study, EB staining revealed the occurrence of significant blood-brain barrier destruction, increased vascular leakage, and tissue edema (Figure 9). The destruction of tight junctions between endothelial cells could be detected due to a decrease in the expression of the tight junction marker protein zonal occludens-1 (ZO-1) in the 3-NP group (Figure 5C, 5D; Figure 9). Interestingly, vascular leakage and the destruction of tight junctions were notably reduced in the C16+Ang-1 group, suggesting that C16+Ang-1 treatment could inhibit the 3-NP-induced effects (Figure 9).

**Figure 10:**
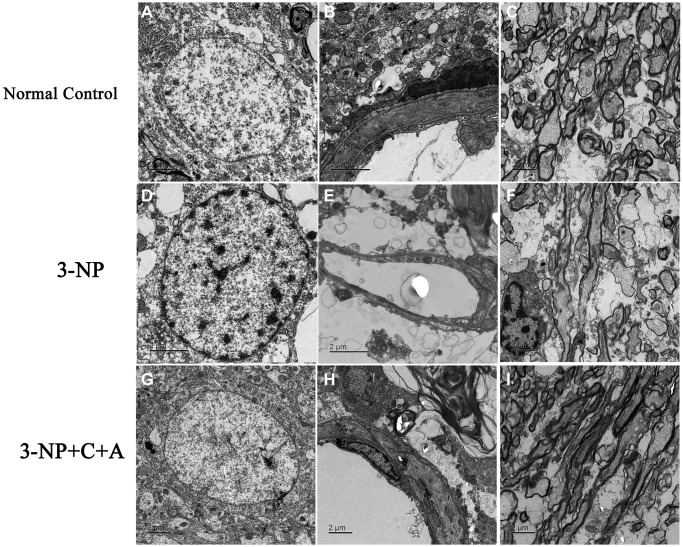
**Treatment with C16+Ang-1 reduces neuronal apoptosis, vascular leakage and blood-brain barrier permeability.** (**A**–**C**) Electron micrographs showing ultrastructural morphology of the normal group. (**A**) Normal neurons with well-defined nuclei and dispersed chromatin (euchromatin). (**B**) Blood vessels have intact tight junctions. There was no tissue edema or blood vessel leakage. (**C**) Normal myelinated axons. (**D**–**F**) Changes in ultrastructural morphology in the 3-NP-treated group. (**D**) Neuronal apoptosis was evidenced by nuclei with condensed, fragmented, and marginated chromatin against the nuclear envelope compared with normal cells. (**E**) Leakage from severe blood vessels and tissue edema in the extracellular space. (**F**) Myelin sheath splitting, loosening, and fusion, and vacuoles were observed. (**G**–**I**) In the 3-NP+C16+Ang-1 group, the morphology of (**G**) nuclei was relatively normal. (**H**) Perivascular edema and (**I**) myelin sheath splitting were reduced.

Next, we examined the brain tissue ultrastructure morphology using electron microscopy. We observed normal nuclei morphology, evidenced by well-defined nuclei with dispersed chromatin. The absence of tissue edema and vascular leakage as well as intact tight junctions was found in the control group (Figure 10A–10C). In the 3-NP group, we noted strong chromatin condensation and marginated chromatin along the nuclear envelope, which are typical features of neuronal apoptosis. Splitting, loosening, and fusion of the myelin sheath, vacuoles, severe vascular leakage, and tissue edema in the surrounding extracellular space were also observed (Figure 10D–10F). Notably, we observed normal nuclei morphology, as well as reduced myelin sheath splitting and reduced perivascular edema in the 3-NP+C16+Ang-1 group (Figure 10G–10I).

### Treatment with C16+Ang-l can counter 3-NP-induced effects on several signaling pathways

**Figure 11:**
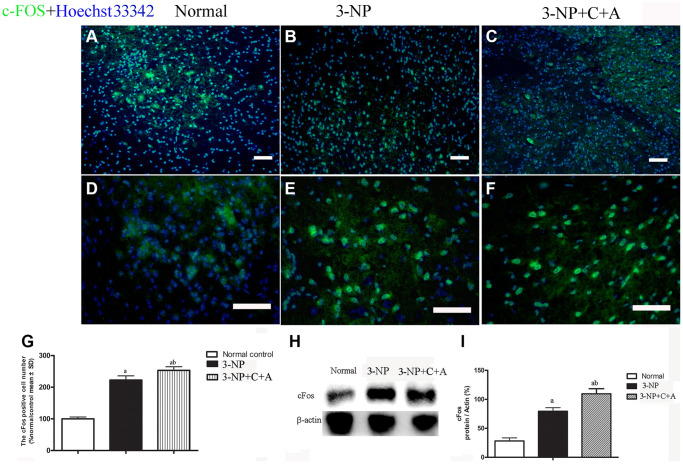
(**A**–**G**): Immunofluorescence staining images of c-FOS (green, immediate early gene, a marker of neuronal activation, (**A**–**C**) at magnification ×100 and (**D**–**F**) at magnification ×200. Cell nuclei were stained with Hoechst33342 dye (blue). (**G**) Semi-quantitative profiles showed elevated c-Fos expression in the 3-NP+vehicle group and a further increase in c-Fos levels with C16+Ang-1 treatment. Scale bar = 100 μm. Western blot images (**H**–**I**) of the levels of c-Fos in the control, 3-NP, and 3-NP+C16+Ang-1 groups showed a similar trend. ^a^*P* < 0.05 versus control; ^b^*P* < 0.05 versus 3-NP-treated mice.

**Figure 12:**
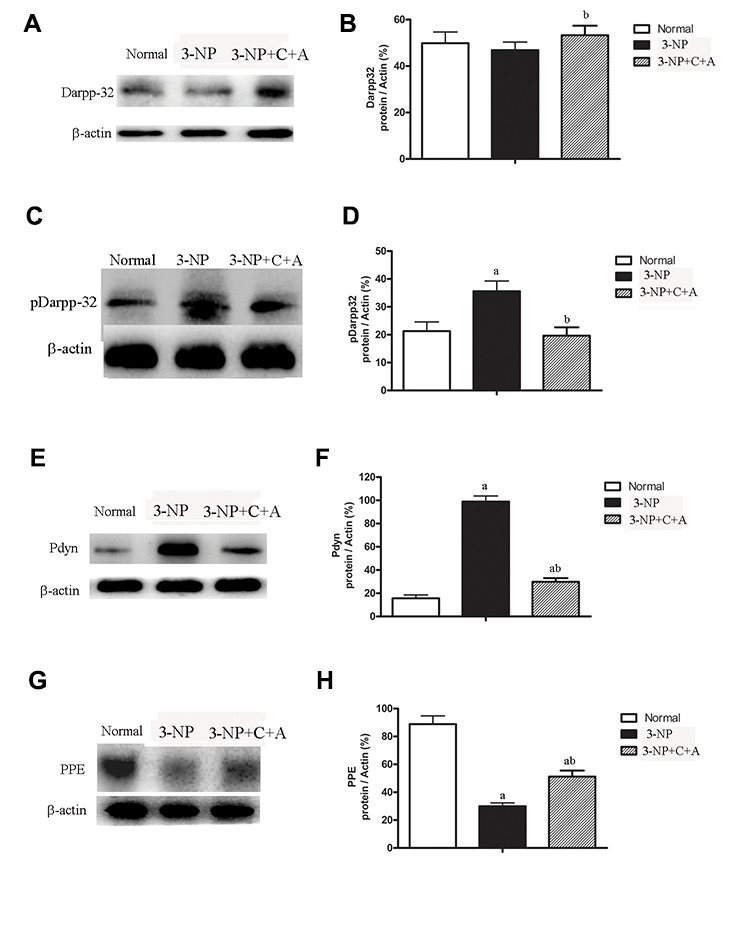
**Significantly elevated pDARPP-32 expression was noted in the 3-NP group, but C16+Ang-1 treatment reduced pDARPP-32 expression.** The PPE and Pdyn expression levels are indicators of imbalances between the striatopallidal and striatonigral pathways. 3-NP insult can increase Pdyn but decrease PPE in the striatum of mice in the 3-NP group. C16+Ang-1 treatment decreased Pdyn but increased PPE expression, suggesting that its mechanism of action likely involved protecting the indirect basal ganglia pathway and the PPE medium spiny neuronal terminals. Western blot images of the levels of (**A**, **B**) DARPP-32, (**C**, **D**) pDARPP-32, (**E**, **F**) Pdyn, and (**G**, **H**) PPE in the control, 3-NP, and 3-NP+C16+Ang-1 groups. ^a^*P* < 0.05 versus control; ^b^*P* < 0.05 versus 3-NP-treated mice.

We observed increased expression of the immediate early gene c-Fos, a marker of neuronal activation, and increased c-Fos expression in the 3-NP+vehicle group; there was also a further upregulation in c-Fos levels with C16+Ang-1 treatment (Figure 11). A slight increase in DARPP-32 expression was observed in all treatment groups (Figure 12A–12B). However, significantly elevated pDARPP-32 expression was noted in the 3-NP group, but C16+Ang-1 treatment reduced pDARPP-32 expression (Figure 12C–12D). The PPE and Pdyn expression levels are indicators of imbalances between the striatopallidal and striatonigral pathways [13]. Using western blotting, we observed higher prodynorphin (Pdyn) but lower preproencephalin (PPE) in the striatum of mice in the 3-NP group, but C16+Ang-1 treatment alleviated the 3-NP-induced effects (Figure 12E–12H).

## DISCUSSION

Administration of 3-NP in the present study induced motor functional impairment, memory loss, abnormal agonist/antagonist muscle co-contraction, axonal loss, inflammation, neuronal apoptosis and autophagy, synaptic vesicles loss and neurotransmitter imbalance, indicating the successful establishment of a dystonia mouse model. In addition, we noted increased permeability of the blood-brain barrier, tissue edema, and the recruitment of inflammatory cells into target tissues. Our observation of 3-NP-induced inflammation in the dystonia mouse model confirmed the previously reported association between inflammation and 3-NP-induced neurodegeneration [7], and was also consistent with prior reports of the significant increase in proinflammatory markers (tumor necrosis factor (TNF)-α, IL-6, and IL-1β) upon 3-NP treatment [8, 20]. In addition to its role in reducing endothelial permeability [16], Ang-1 also has reported roles in reducing LPS-induced microvascular dysfunction and endotoxin-induced vascular leakage in the CNS, thereby inhibiting inflammatory cell infiltration [21]. Given that the combined treatment of Ang-1 and C16 could synergistically inhibit inflammation [21], we explored the efficacy of C16+Ang-1 as a treatment option for dystonia by assessing the recovery of neuronal morphology and motor functions using an array of histological, immunohistochemical, biochemical, and electrophysiological experiments. We showed that C16+Ang-1 treatment effectively attenuated severe inflammation within the CNS and improved mitochondrial functional impairment, suggesting the neuroprotective potential of C16+Ang-1 application in dystonia therapy.

Neuronal apoptosis and inflammation as well as decreased ATP production in the corpus striatum and nigra substance areas are characteristic of 3-NP neurotoxicity [22]. Apoptosis induction is correlated with high expression of pro-inflammatory factors, oxidative stress-induced mitochondrial dysfunction, and ROS production [22]. Antioxidant compounds have been found to improve mitochondrial function in HD by reducing oxidative stress and blocking oxidative stress-induced apoptosis and inflammation [23, 24]. Similarly, reductions in the expression pro-inflammatory cytokine release, ROS production, apoptotic marker active caspase-3 and autophagy marker IC3 levels upon C16+Ang-1 treatment in this study supported the alleviation of neurotoxicity in HD-like dystonia mice.

Since c-Fos is a neuronal activation marker, its upregulation could be due to injury or disturbance of neural networks [25]. However, the neuroprotective effects can also lead to neuronal activation [26]. For instance, c-Fos itself could be neuroprotective; hence, its upregulation might be a protective reaction of cells upon injury [27]. Consistent with this report, we noted increased c-Fos levels upon 3-NP treatment. The Ang-1-mediated neuroprotective effects observed in this study were in line with the reported role of Ang-1 in stimulating c-Fos expression through promoting the phosphorylation of extracellular signal-regulated kinases (ERK)1/2, phosphoinositide 3-kinase (PI3K), protein kinase B (Akt), and glycogen synthase kinase (GSK)-3β [28]. Indeed, we noted further upregulation in c-Fos levels with C16+Ang-1 treatment, providing further support for Ang-1’s neuroprotective ability [29].

DARPP-32, which is abundant in the medium spiny neurons of the striatum in the basal ganglia, is converted into an inhibitor of protein phosphatase-1 upon cAMP-dependent protein kinase (PKA)-catalyzed phosphorylation. The dopamine-mediated effect on motor behavior via the cAMP signaling cascade was found to be augmented by DARPP-32 phosphorylation in medium spiny neurons [30]. Hence, the elevated pDARPP-32 levels in our dystonia mice may be a consequence of long-term modifications.

Follicle-stimulating hormone (FSH) could reportedly act as the primary survival factor to reduce 3-NP-induced and ROS-induced apoptosis by inhibiting the expression of p53 upregulated modulator of apoptosis (PUMA) [31]. The PI3K/Akt inhibitor LY294002 could reverse FSH-induced downregulation of PUMA and increase apoptosis, indicating the involvement of the PI3K/Akt pathway in cell survival [31].

In the present study, reduced PPE but increased Pdyn levels in the striatum indicated the disruption of the indirect basal ganglia pathway that regulates the selection accuracy of learned motor actions [32]. PPE medium spiny neuronal terminals in the caudate nucleus of HD brains are more susceptible to P neuronal damage [33]. Reduced excitability of inhibitory circuits in the motor cortex and disrupted basal ganglia input could be the underlying cause of dystonia in our animal model [34].

Alternative pathways including the α5β1-PI3K-Bcl-2 and α5β1/αvβ3-integrin-dependent Akt, ERK, and c-Jun N-terminal kinase (JNK) signaling pathways are involved in αvß3 and/or α5β1-integrin-mediated PI3K/Akt activation as well as the modulation of neuronal survival [28]. Our observations from dystonia mice were consistent with previous reports of neuroprotection upon PI3K/Akt activation by C16 and Ang-1, alone and in combination, which promoted cell viability and suppressed apoptosis and autophagy in basal ganglia neurons and terminals in the indirect pathway [29, 35].

**Figure 13:**
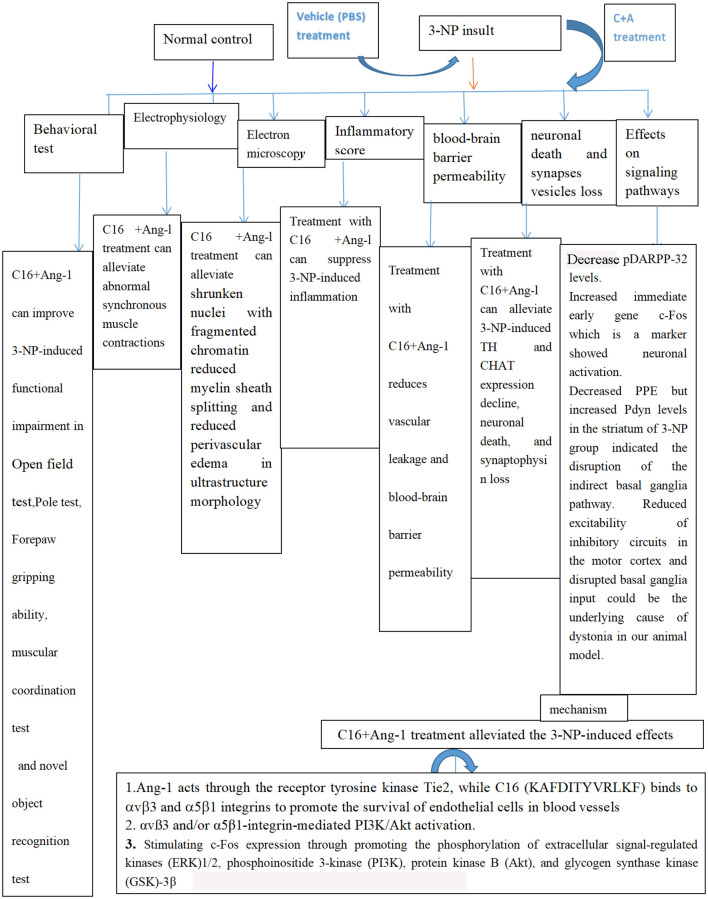
Schematic diagram showing all protective effects of combined C16 and Ang-1 treatment in 3-NP-induced mice in this study.

In conclusion, given the association between neuroinflammation and neurodegenerative diseases such as HD [36], preventing inflammatory cell transmigration and improving vascularity could ameliorate inflammation-related CNS diseases (Figure 13). As the effects of C16+Ang-1 were shown to be non-specific, these compounds could be adopted as complementary therapeutics against different neuroinflammatory diseases. Efforts in dosage determination and the characterization of side effects are ongoing in our laboratory and we aim to investigate the feasibility of C16+Ang-1 as a combination therapeutic in other neuroinflammatory disease models.

## MATERIALS AND METHODS

### Drugs

Ang-1 with a purity level >90% (peptide sequence: KLENYIVENMKSEMAQIQQNAVQNHTATMLEIGTSLLSQTAEQTRKLTDVETQVLNQTSRLE IQLLE NSLSTYKLEKQLLQQTNEILKI; synthesized by Shanghai Science Peptide Biological Technology Co., Ltd, China) was dissolved in distilled water to a final concentration of 4 mg/mL. The C16 peptide was dissolved in distilled water with 0.3% acetic acid, passed through a 0.22 μm disc filter, and was pH-adjusted to pH 7.4 with NaOH. An equal volume of sterile phosphate-buffered saline (PBS) was then added to the C16 peptide solution to obtain a final concentration of 2 mg/mL.

### Animal care and grouping

Sixty male C57/BL6 mice (20–25 g; Beijing Vital River Lab Animal Technology Co., Ltd., Beijing, China) were housed in a pathogen-free environment at a temperature of 22 ± 1°C under a 12 h light-dark cycle. Food and water were provided *ad libitum*. All experiments were conducted according to the guidelines for the care and use of laboratory animals specified by the National Institutes of Health (NIH), USA, with approval from the Animal Care Committee of the Chinese Academy of Medical Sciences, China. The 60 mice were randomly divided into three groups (*n* = 20/group): control, 3-NP treatment (3-NP), and 3-NP+C16+Ang-1 treatment (3-NP+C+A).

### Establishment of HD-like dystonia mouse model

Mice in the 3-NP and 3-NP+C16+Ang-1 treatment groups were injected with 3-NP (Sigma Aldrich, St. Louis, MO, USA) in PBS pH 7.4 intraperitoneally (*i.p.*) at 50 mg/kg twice daily for 5 days to induce HD-like dystonic symptoms, while the control group was injected with an equal volume of normal saline (*i.p.*) [2]. Mice in the 3-NP+C16+Ang-1 treatment group were intravenously administered with 0.5 mL drug including 40 μg Ang-1 + 200 μg C16 daily for 2 weeks, one day after 5 days of 3-NP injection. Control and 3-NP mice were also intravenously administered with 0.5 mL PBS via tail vein injections.

### Open field test

Changes in the exploratory behavior and activity of the animals were determined using the open field test (OFT). Each mouse was placed in an enclosure with dimensions of 44 × 44 × 32 cm and its activity was monitored for 5 min under low light (40 W) conditions. Changes in the animals’ positions and movements were monitored using a video camera placed over the enclosure. Measurements of the total distance travelled, mean velocity (V_mean_), and activity were collected and processed using the EthoVision software (The Noldus Community, Wageningen, The Netherlands). The average of three independent experiments was calculated and used for statistical analyses.

### Pole test

A ball (1 cm diameter) was fixed on top of a pole (height 50 cm, diameter 1 cm) wrapped in gauze to prevent the mouse from slipping while climbing up the pole. The mice were placed on the top of the pole, head facing upward. We measured the total time (T-total) taken to climb down the pole, and the time at which the mice turned downward (T-turn). The data for three independent experiments were used for statistical analyses.

### Forepaw gripping ability

Each mouse was trained on a grip strength meter (GSM; TSE-Systems) for 2 days, for 5 min each day. The training involved suspending each mouse by the tail just above the bar of the GSM and pulling it away from the bar by its tail in one smooth motion until its forepaw grip on the GSM bar was released. The force (in grams) at the instant before the mouse’s forepaw grip on the bar was released was measured using the GSM after the 2-day training. Three independent experiments were performed.

### Muscular coordination test

We conducted the rotarod experiment once a week over 8 weeks [20] to study the motor-coordination ability of the mice. Briefly, each mouse was placed on a rotarod (9 cm diameter, 8 cm wide; KN-75, Natsume Seisakusho Co., Ltd) rotating at 15 rpm, and the length of time the mouse remained on the rod before falling off was measured. Three independent experiments were performed. The mice were trained two or three times a week on the rotarod before each experiment.

### Novel object recognition (NOR) test

The NOR test was performed to assess the animal's ability to recognize a novel object in its environment, which is a function associated with declarative memory that allows the conscious recall of facts and events. The difference in the exploration time between novel and familiar objects was calculated. We conducted the NOR test using a rectangular glass-fronted enclosure with dimensions of 60 × 40 × 50 cm in a soundproof room under low light conditions. The test consisted of three phases: habituation, familiarization, and testing, each on separate days. In the habituation phase, each mouse was given 20 min to explore the enclosure devoid of objects before returning it to its holding cage. In the familiarization phase, each mouse was given 5 min to explore the enclosure, which contained two identical sample objects (A). Subsequently, after 90 min, the mouse was returned to the enclosure, which contained two different sample objects (A + B), and was given 5 min to explore. In the test phase, 24 h after the familiarization phase, the mouse was returned to the enclosure containing two objects, one identical to the sample object A and the other a novel object (A + C), and was given 5 min to explore. We evaluated the long-term memory in this phase. The length of time each animal spent near the object in the novel location (NL) and near the familiar object (F) were recorded to determine the preference for novelty. The discrimination score was calculated using (NL–F)/ (NL+F) [35].

### Electrophysiology

Electrophysiological experiments were carried out 1 week following 3-NP induction using a telemetry-based two-lead electromyography (EMG) transmitter (F20-EET, Data Sciences International^®^, St. Paul, MN). The skin of the animal’s hind limbs was separated from the muscle layer and transmitter electrodes were attached to both hind limbs through the quadriceps femoris and biceps femoris muscles, and the incision made to insert the electrodes was sutured. Using an acupuncture pin electrode to initiate a stimulus in the quadriceps femoris can reduce non-specific signal contamination [22]. EMG data were collected using the DataQuest Acquisition hardware (Data Sciences^®^ International, St. Paul, MN) and were exported to LabChart (ADInstruments, Colorado Springs, CO) for further analysis.

### Perfusion and tissue processing

Mice in the normal, 3-NP and 3-NP+C16+Ang-1-treated groups (10 mice/group) were sacrificed at 4 weeks post-induction. The brain of each mouse was harvested and half of it was fixed in 4% paraformaldehyde for 4 h before immersing in a PBS solution with 30% sucrose. Coronal sections (20 μm-thick) of the corpus striatum and nigra substance regions were obtained using a freezing microtome and a Leica cryostat (Buffalo Grove, IL, USA) and mounted onto 0.02% poly-L-lysine-coated slides for subsequent histology experiments [17–19, 35].

### Transmission electron microscopy

Half of the striatum and nigra substance was fixed in a 2.5% glutaraldehyde solution, washed three times with 0.1 M PBS, and soaked in 1% osmium tetroxide at 4°C overnight. After washing three times with 0.1 M PBS, the sections were examined under a transmission electron microscope (TEM, JEOL Ltd. Tokyo, Japan) as previously described [17–19, 35].

### Hematoxylin and eosin (HE) staining

Inflammatory cell infiltration in HE stained mouse brain tissue sections of the striatum and nigra substance was examined under a light microscope [17–19, 35] by an experienced pathologist without prior knowledge of the experimental conditions. Cells in five random fields of view were counted for each section. The severity of infiltration was scored as follows: 0, no inflammation; 1, infiltration around blood vessels and meninges only; 2, mild infiltration in parenchyma tissue (1–10/section); 3, moderate infiltration in parenchyma tissue (11–100/section); and 4, severe infiltration in parenchyma tissue (100/section). Neurons in each group were visualized under a Nikon TE-300 microscope (Nikon, Japan) at ×200 magnification and the percentage (%) of neuronal survival was calculated (with respect to the % of the sham control).

### Evans Blue (EB) staining

Rat blood-brain barrier permeability was assessed using the EB extravasation method with modifications [16]. Briefly, sodium pentobarbital (60 mg/kg, *i.p.*)-anesthetized mice (*n* = 5/group) were sacrificed at 4 weeks post-induction via right femoral vein EB dye (4 mL/kg) infusion at 37°C over 5 min for 2 h. Saline (300 mL) perfusion of the EB-stained tissue sections (20-μm-thick) was then performed to wash out the remaining dye before visualization under a red ultraviolet light filter and analysis using the Image J software (NIH, Bethesda, MD, USA). Half of the tissue sections were homogenized in 750 μL of *N, N-*dimethylformamide (DMF, Sigma, St. Louis, MO) in the dark at room temperature for 72 h before centrifugation at 10,000 × *g* for 25 min. Dye concentrations (μg/g of tissue weight) in the supernatant were measured using a spectrophotometer (Molecular Devices OptiMax, USA) at 610 nm and calculated against a standard curve [16].

### Immunofluorescence staining

As previously described [17–19, 35], the tissue sections were permeabilized and blocked with 0.3% Triton X-100/10% normal goat serum in 0.01 M PBS for 30 min before incubating with rabbit/mouse synaptophysin (Syn; 1:500; Abcam, Cambridge, MA, USA), prodynorphin (Pdyn) and preproencephalin A (PPE) (1:500, Thermo Fisher Scientific, Waltham, MA, USA), tyrosine hydroxylase (TH) and choline acetyltransferase (CHAT 1:500; Abcam, Cambridge, MA, USA), c-Fos (1:200; Abcam, Cambridge, MA, USA), dopamine- and cAMP-regulated phosphoprotein of 32 kDa (DARPP-32) and phosphorylated-DARPP-32 (Thr34; pDARPP-32) (1:500; R&D Systems, Minneapolis, MN, USA), cluster of differentiation 38 (CD38) and CD206 (1:200; Santa Cruz Biotechnology) and ZO-1 (1:500; Cayman Chemical, Ann Arbor, MI, USA), microtubule-associated protein light chain 3B (LC3B) (1:200, Novus Biologicals, Littleton, CO, USA) and caspase 3 (1:500; Cayman Chemical, Ann Arbor, MI, USA) primary antibodies overnight at 4ºC. Sections were rinsed three times with PBS before incubation with fluorescein isothiocyanate (FITC)/Trimolecular Fluorescence Complementation (TRIFC)–conjugated goat anti-rabbit/mouse secondary antibody (1:200; Invitrogen, CA) for 1 h at 37°C and then mounted with Antifade Gel/Mount aqueous mounting media (Southern Biotech). Three visual fields per section (5 sections) of the corpus striatum and nigra substance of each animal were randomly selected for counting and imaged under an Olympus BX61 fluorescence microscope (×200 magnification). Image analysis was performed using the NIH image processing software (NIH, Bethesda, MD, USA).

### Enzyme-linked immunosorbent assay (ELISA)

ELISA was performed to quantify the cytokine levels. Mice (*n* = 5 per time point per group) were sacrificed by decapitation at 3 and 8 weeks post-induction and peripheral blood was collected at 4°C. Heparin was added to samples to prevent coagulation. The blood samples were first centrifuged at 1,000 × g for 20 min, then at 10,000 × g for 10 min at 4°C, to ensure complete platelet removal. Plasma samples were first incubated in 96-well plates precoated with interleukin 6 (IL-6), reactive oxygen species (ROS), and γ-aminobutyric acid (GABA; R&D Systems, Minneapolis, MN, USA) primary antibodies for 1 h at 37°C, and then were subsequently incubated with horseradish peroxidase (HRP)-conjugated goat anti-rabbit IgG secondary antibody (1:2000; Biorad, CA, USA) for 1 h at 37°C. The concentration of bound proteins was measured using a microplate reader (model 680, Bio-Rad Laboratories, Corston, UK) at 450 nm and analysis was performed using the GraphPad Prism version 4 software (GraphPad Prism Software, Inc, CA, USA).

### Western blot analysis

Mice (*n* = 5 per time point per group) were sacrificed by decapitation and their striatum and nigra substance were harvested at 2 and 8 weeks post-induction. Total protein was isolated from tissue lysates and then the proteins were separated by 12% SDS-PAGE electrophoresis. Protein bands were transferred onto a polyvinylidene difluoride membrane and probed with rabbit Syn (1:1500; Abcam, Cambridge, MA, USA), Pdyn and anti-PPE (1:1000, Thermo Fisher Scientific, Waltham, MA, USA), TH and CHAT (1:1500; Abcam, Cambridge, MA, USA), c-Fos (1:1000; Abcam, Cambridge, MA, USA), pDARPP-32 (Thr34) and DARPP-32 (1:800; R&D Systems, Minneapolis, MN, USA), CD38 and CD206 (1:500; Santa Cruz Biotechnology), ZO-1 (1:1000; Cayman Chemical, Ann Arbor, MI, USA), LC3B (1:500, Novus Biologicals, Littleton, CO, USA), and caspase 3 (1:1000; Cayman Chemical, Ann Arbor, MI, USA) primary antibodies for 12 h at room temperature. The membranes were then incubated with HRP-conjugated goat anti-rabbit secondary antibody (1:5000; Santa Cruz, CA, USA) for 1 h before band visualization using the ECL Plus detection system (Amersham Pharmacia Biotech) and imaging. Rabbit anti-β-actin antibody (1:5000; Abcam, MA, USA) was used as the internal normalization control.

### Statistical analysis

Statistical analysis was performed using the SPSS 13.0 software (Chicago, IL, USA) and GraphPad Prism version 4.0. Data were analyzed using the Kruskal-Wallis nonparametric one-way analysis of variance (ANOVA) followed by post hoc analysis and were presented as the mean ± standard deviation (SD). Values of *P* < 0.05 were considered statistically significant.

### Data availability statement

The datasets used and/or analyzed during the current study are available from the corresponding author on reasonable request.
